# Effects of Cooking Methods on the Flavor Characteristics of Volatiles of *Boletus edulis* Based on GC‐IMS, E‐Nose, and Sensory Evaluation

**DOI:** 10.1002/fsn3.71430

**Published:** 2026-01-14

**Authors:** Xin Wu, Jingfa Wang, Huizhen Liu, Fanjun Sun, Fucan Liu, Jing He, Furong Tian, Chunxia Gan

**Affiliations:** ^1^ School of Tourism Kunming University Kunming Yunnan Province People's Republic of China; ^2^ Faculty of Modern Tourism and Catering Industry Kunming Yunnan Province People's Republic of China

**Keywords:** *Boletus edulis*, cooking methods, E‐nose, GC‐IMS, sensory evaluation, volatiles

## Abstract

This study investigated the changes in volatile flavor compounds of *Boletus edulis* under different cooking methods (fresh, steaming, frying, roasting and boiling) using gas chromatography‐ion mobility spectrometry (GC‐IMS) and electronic nose technology. Characteristic fingerprints of flavor compounds were established, and principal component analysis (PCA) was applied for quantitative analysis of flavor substance changes, combined with a sensory evaluation system to comprehensively assess the impact of cooking processes on the volatile flavor of *B. eduli*s. A total of 49 volatile compounds were detected in the samples of *B. edulis* subjected to five different processing methods. These include 27 thioethers, 16 aldehydes, 12 alcohols, 10 ketones, 1 ester, 4 pyrazines, 2 furans, 2 acids, and 2 heterocyclic compounds. Flavor fingerprint analysis revealed significant compositional differences among groups: Fresh *B. edulis* (NG0) had higher signal intensities of 2‐octenal, 1‐octen‐3‐one, and 3‐octanone; steamed samples (NG1) showed weakened signals of these three compounds but enhanced 1‐octen‐3‐ol and heptanal; fried samples (NG2) exhibited stronger signals of 2‐furaldehyde, 1‐octen‐3‐ol and other compounds; roasted samples (NG3) had prominent signals of 1‐octen‐3‐one, 2‐methyl‐2‐hepten‐6‐one, etc.; boiled samples (NG4) were characterized by high signals of 2‐propanone, 1‐octen‐3‐ol, etc. Fresh, steamed, and boiled *B. edulis* mainly contained E‐2‐octenal, 1‐octen‐3‐one, and other compounds, while high‐temperature cooking (frying, roasting) led to flavor compounds dominated by aldehydes and pyrazines. The electronic nose detection effectively discriminated among *B. edulis* samples prepared with different cooking methods. From the perspective of the comprehensive sensory evaluation scores, fried and roasted *B. edulis* obtained higher scores and demonstrated a more well‐balanced flavor profile, suggesting that frying and roasting might be the optimal cooking methods for *B. edulis*. These results provide a theoretical basis for flavor regulation during *B. edulis* cooking, data support for the improvement and innovation of *B. edulis* dishes, and technical guidance for the development of *B. edulis* processed products.

## Graphical abstract

This study successfully established characteristic fingerprints of volatile flavor components in *Boletus edulis* subjected to different cooking methods. Using principal component analysis (PCA) to quantitatively evaluate changes in flavor substances, and combined with a sensory evaluation system, it comprehensively assessed the impact of cooking processes on the volatile flavor characteristics of *B. edulis*. All complementary analytical techniques effectively differentiated samples by cooking method, with comprehensive analysis identifying frying and roasting as the optimal processing methods for enhancing *B. edulis*'s volatile flavor characteristics.

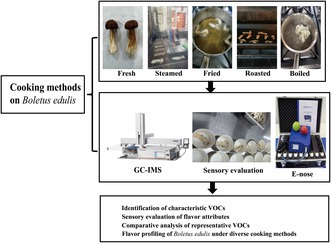

## 1 Introduction

*Boletus edulis* is distributed worldwide and is an important rare wild edible mushroom. China boasts abundant *B. edulis* resources, particularly in Yunnan. With its extensive mountainous terrain and predominantly hot and humid climate, Yunnan provides a unique growth environment for *B. edulis*. This mushroom is not only rich in nutrients but also possesses extremely high medicinal value, including immune regulation, anti‐tumor, anti‐oxidation, anti‐inflammatory, and anti‐fatigue properties (Wang et al. 2021). It is a rare edible fungus with dual medicinal and culinary value. Currently, *B. edulis* is predominantly used as a cooking ingredient, and its application is gradually expanding to beverages, condiments, and functional foods. Cooking methods include dry‐heat cooking (e.g., baking and grilling), moist‐heat cooking (e.g., stewing, braising, and boiling), steam cooking (e.g., steaming), and frying (e.g., deep‐frying, stir‐frying, and pan‐frying). Common cooking methods encompass stir‐frying, steaming, boiling, deep‐frying, and stewing, among others. When it comes to *B. edulis*, common cooking methods such as stir‐frying, roasting, steaming, and boiling can each highlight distinct flavors, allowing the characteristic aroma and rich texture of mushrooms to unfold in diverse ways.

The flavor of food refers to the comprehensive sensory experience involving the taste buds, olfactory receptors, visual perception, and tactile nerves once the food enters the mouth. Volatile compounds play a fundamental role in flavor perception. A study by Misharina et al. (2009) found that unsaturated alcohols and ketones containing eight carbon atoms not only determine the aroma profile of raw mushrooms but also contribute to the formation of the aroma in cooked mushrooms. Variations in temperature, heat transfer modes, and heating media lead to the fact that different cooking methods bring about unique impacts on food aroma (Bassam et al. 2022).

In a study by Zhou et al. (2022), the influence of various cooking methods—including boiling, steaming, microwaving, deep‐frying, and pressure cooking—on the nutritional composition, antioxidant activity, and both volatile and non‐volatile flavor‐active compounds was investigated using three varieties of Lentinus edodes originating from Guizhou Province. The findings demonstrated that microwaving contributed to an increase in bioactive compounds and enhanced antioxidant activity, while also effectively preserving non‐volatile flavor compounds. In contrast, pressure cooking and deep‐frying were established as the most effective techniques for augmenting flavor compounds. In a study by Xie et al. (2024), the influence of various cooking methods on the volatile aroma compounds of *Agaricus bisporus* was examined. It was observed that baking played a significant role in the formation of flavor compounds, imparting a distinct flavor profile to the mushrooms. In contrast, the steamed samples exhibited an odor highly similar to that of the fresh group. The overall aroma of food is predominantly shaped during cooking operations through key mechanisms such as the Maillard reaction, lipid oxidation, amino acid degradation, and their intricate interplay (Flaskerud et al. 2017; Li et al. 2022; Shakoor et al. 2022). Over an extended period, sensory evaluation has served as the predominant approach for analyzing and assessing how cooking methods and conditions influence food flavor. Trained sensory panelists perform these assessments following standardized sensory evaluation protocols. While conventional sensory analysis can accurately capture consumers' subjective experiences, it suffers from notable drawbacks. For example, sensory evaluators require extensive and systematic training, and are highly susceptible to fatigue during prolonged sessions. In response to these limitations, a range of sensing and detection technologies have been developed and are being progressively adopted for the analysis of volatile organic compounds (VOCs). These emerging techniques include headspace solid‐phase microextraction combined with gas chromatography–mass spectrometry (HS‐SPME‐GC–MS), headspace gas chromatography–ion mobility spectrometry (HS‐GC‐IMS), electronic nose (E‐nose), and colorimetric sensor array (CSA)‐based olfactory visualization systems (Liu et al. 2024; Wang et al. 2019; Yang et al. 2021; Yu et al. 2022).

Cao 2012 analyzed and identified the flavor compounds of *B. edulis* using gas chromatography–mass spectrometry combined with the retention index method and gas chromatography‐olfaction measurement technology. The results indicated that among the 53 identified volatile flavor compounds of *B. edulis*, 29 compounds contribute to its flavor. The major contributors include acetaldehyde (imparting a grassy flavor), methylpyrazine (with a nutty flavor), 3‐methylthiopropionaldehyde (giving off a burnt aroma), 2,6‐dimethylpyrazine (producing a barbecue aroma), 1‐octen‐3‐ol (contributing to the mushroom flavor), 2‐pentylfuran (exhibiting a bean‐like aroma), 2‐ethyl‐6‐methylpyrazine (generating a roasted aroma), 3‐octenedione (associated with the mushroom flavor), phenylacetaldehyde (providing a floral aroma), and other compounds (Cao 2012). In Li's research, the flavor profiles of the pileus and stipe of *B. edulis* from eight distinct geographical origins were evaluated using electronic nose (E‐nose), headspace gas chromatography–ion mobility spectrometry (HS‐GC‐IMS), and headspace solid‐phase microextraction gas chromatography–mass spectrometry (HS‐SPME‐GC–MS). A total of 23 key volatile organic compounds (VOCs) with odor activity values (OAVs) exceeding 1 were characterized, along with the simultaneous identification of 19 aroma categories. Vegetable‐like and earthy aromas were identified as the predominant notes across all pileus and stipe samples. Additionally, balsamic and musty aromas were recognized as the principal and distinctive characteristics of the pileus. (Li et al. 2024).

Currently, studies on the flavor of *B. edulis* are primarily focused on the effects of freshness, packaging, storage conditions, and food processing, with limited attention given to how cooking methods influence its flavor compounds. In this investigation, gas chromatography‐ion mobility spectrometry (GC‐IMS) and electronic nose (E‐nose) were utilized to examine changes in the volatile flavor profiles of *B. edulis* subjected to various cooking techniques. A characteristic flavor fingerprint was subsequently constructed. Principal component analysis (PCA) was applied to interpret variations in flavor constituents, and sensory evaluation was further incorporated to comprehensively assess the impact of different cooking methods on the volatile flavor characteristics of *B. edulis*.

## 2 Materials and Methods

### 2.1 Materials

1500 g Fresh, worm‐free *B. edulis* were purchased from Yunnan Mushuihua Wild Mushroom Trading Center.

### 2.2 Cooking Methods

The cooking conditions for the *B. edulis* samples were established primarily based on traditional Chinese culinary practices, with adjustments informed by preliminary experimental results. The final optimal parameters were established via sensory evaluation carried out in a professional Chinese kitchen setting. All samples were processed immediately following cooking prior to subsequent analysis.

#### 2.2.1 Steam Cooking

Fresh and worm‐free *B. edulis* were cut into 2 mm slices with a knife. When the water in the steamer started boiling, the mushroom slices were put into the steamer and steamed for 10 min. Then, they were taken out and allowed to cool.

#### 2.2.2 Frying Cooking

Fresh and worm‐free *B. edulis* were cut into slices about 2 mm thick with a knife. The soybean oil was heated to 160°C, and the *B. edulis* slices were fried for 5 min with a ratio of *B. edulis* to oil being 1:1. During the frying process, the slices were constantly turned. After frying, absorbent paper was used to remove the excess oil from the *B. edulis* slices.

#### 2.2.3 Boiled Cooking

Fresh and worm‐free *B. edulis* were manually sectioned into 2 mm thick slices using a blade. The slices were subsequently subjected to boiling in water for 30 min, after which they were retrieved and allowed to cool.

#### 2.2.4 Roasted Cooking

Fresh and intact *B. edulis* specimens were sliced to approximately 2 mm thickness using a manual blade. An electric oven (DKX‐F10R6, Bear Electric Appliance Co. Ltd.) was preheated to 180°C prior to roasting. The sliced samples were then transferred to the preheated oven and roasted for 30 min, after which they were removed and allowed to cool to ambient temperature.

### 2.3 GC‐IMS Analysis

After sample treatment, accurately weigh 3 g of each sample and place it in a 20 mL headspace vial. Incubate at 60°C for 15 min before injection. Each sample is measured in triplicate. The raw *B. edulis* samples are labeled as NG0, the steamed *B. edulis* samples are labeled as NG1, the fried *B. edulis* samples are labeled as NG2, the roasted *B. edulis* samples are labeled as NG3, and the boiled *B. edulis* samples are labeled as NG4. The headspace injection was performed under the following conditions: an incubation temperature of 60°C, an incubation time of 15 min, a needle temperature of 85°C, an injection volume of 500 μL, and an agitation speed of 500 rpm.

The gas chromatography (GC) analysis was conducted under the following conditions: a MXT‐WAX capillary column (15 m × 0.53 mm × 1 μm; Restek Corporation, USA) was used, with the column temperature maintained at 60°C. The carrier gas flow rate was initially set to 2 mL/min for 2 min, increased from 2 to 100 mL/min over the period from 2 to 20 min, and then held constant at 100 mL/min until 30 min.

For ion mobility spectrometry (IMS), the parameters were configured as follows: a drift gas flow of 150 mL/min was employed; both the carrier and drift gas consisted of nitrogen (N_2_); the total analysis time was set to 30 min, and the IMS system was maintained at a temperature of 45°C (Bi et al. 2023).

### 2.4 Data Processing

A mixed standard consisting of six ketones was analyzed, and a calibration curve was constructed to correlate retention time with the retention index. The retention index of each substance was subsequently determined according to the retention time of the target compound. For qualitative identification, the GC retention index database (NIST 2020) and the IMS migration time database integrated within the VOCal software were employed for library matching and comparative analysis.

Using plug‐in tools within the VOCal data processing software—including Reporter, Gallery Plot, and Dynamic PCA—three‐dimensional spectra, two‐dimensional spectra, difference spectra, fingerprint maps, and PCA plots of volatile compounds were generated. These visualizations facilitated the comparative analysis of volatile organic compounds across samples.

OPLS‐DA was performed using SIMCA 14.1 (Umetrics AB, Umeå, Sweden) with Pareto (Par) scaling. Supervised regression modeling based on OPLS‐DA was applied to derive variable importance in projection (VIP) values. Differential aroma compounds were identified based on a significance threshold of *p* < 0.05 and a VIP score exceeding 1.

### 2.5 E‐Nose Measurement

The flavor characteristics of *B. edulis* were analyzed with slight modifications based on the method described by Xie et al. (2024). Samples of *B. edulis* under different treatments (5.00 g each) were transferred into 50 mL centrifuge tubes and equilibrated at room temperature for 30 min prior to analysis. The electronic nose system (PEN3 Airsense, Schwerin, Germany) was employed, which incorporates an array of 10 metal oxide gas sensors: W1C (aromatics), W5S (nitrogen oxides), W3C (ammonia and aromatics), W6S (hydrogen), W5C (alkanes and aromatics), W1S (short‐chain alkanes), W1W (inorganic sulfur), W2S (alcohols/ethers/aldehydes/ketones), W2W (organic sulfur), and W3S (long‐chain alkanes). The operational parameters for the electronic nose were set as follows: a sampling interval of 1 s per group, sensor self‐cleaning duration of 60 s, sample injection time of 5 s, injection flow rate of 400 mL/min, and total analysis sampling time of 80 s, with data collected between 69 and 71 s used for further compilation and analysis. All samples were analyzed in triplicate, and the mean values were utilized in subsequent data processing.

### 2.6 Sensory Evaluation of Different Boletus Eduli Samples

A sensory evaluation was carried out on the raw *B. edulis* samples (NG0) as well as those that underwent steaming (NG1), frying (NG2), roasting (NG3), and boiling (NG4). The sensory evaluation panel consisted of five males and five females, all aged between 18 and 30, and affiliated with Kunming University; all sensory panelists were trained. The characteristic aromas chosen for the sensory evaluation were determined based on previously published studies (Qian et al. 2018; Zhang et al. 2018).

## 3 Results Discussion

### 3.1 Comparison of the 2D and 3D Spectra of *B. edulis* From Different Cooking Treatments

**FIGURE 1:**
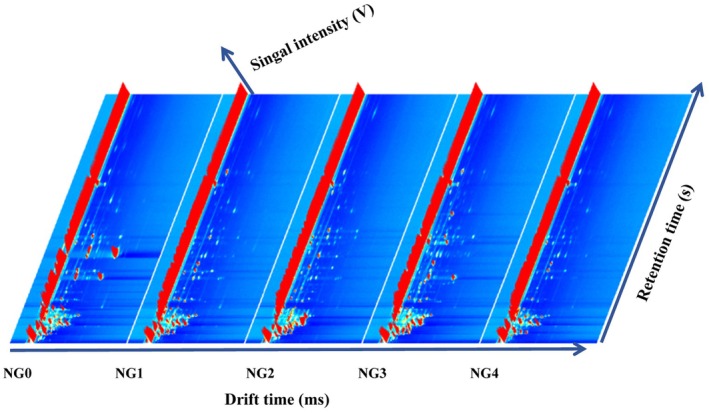
The three‐dimensional spectrum of volatile compounds in the sample. NG0, Fresh *Boletus edulis*; NG1, steamed *B. edulis*; NG2, fried *B. edulis*; NG3, roasted *B. edulis*; NG4, boiled *B. edulis*.

The volatile compounds of steamed, fried, boiled, and roasted *B. edulis* were characterized using gas chromatography‐ion mobility spectrometry (GC‐IMS), yielding a three‐dimensional spectral profile of the volatile compounds (Figure 1). In the resulting spectrum, the *x*‐axis corresponds to the ion migration time, the *y*‐axis to the retention time, and the *z*‐axis to the signal intensity. From left to right, the samples are arranged as follows: fresh (NG0), steamed (NG1), fried (NG2), roasted (NG3), and boiled (NG4). As can be observed from the figure, the content of volatile compounds in the fried and roasted samples was significantly higher than that in the samples prepared by the other two cooking methods. Meanwhile, the steamed and boiled samples exhibited comparable contents of volatile compounds, likely resulting from variations in the loss or formation of these compounds due to different cooking methods applied to *B. edulis*. In view of the relatively elementary representation offered by the 3D spectrum, the two‐dimensional (2D) spectral data of the volatile compounds were integrated for more detailed examination.

**FIGURE 2:**
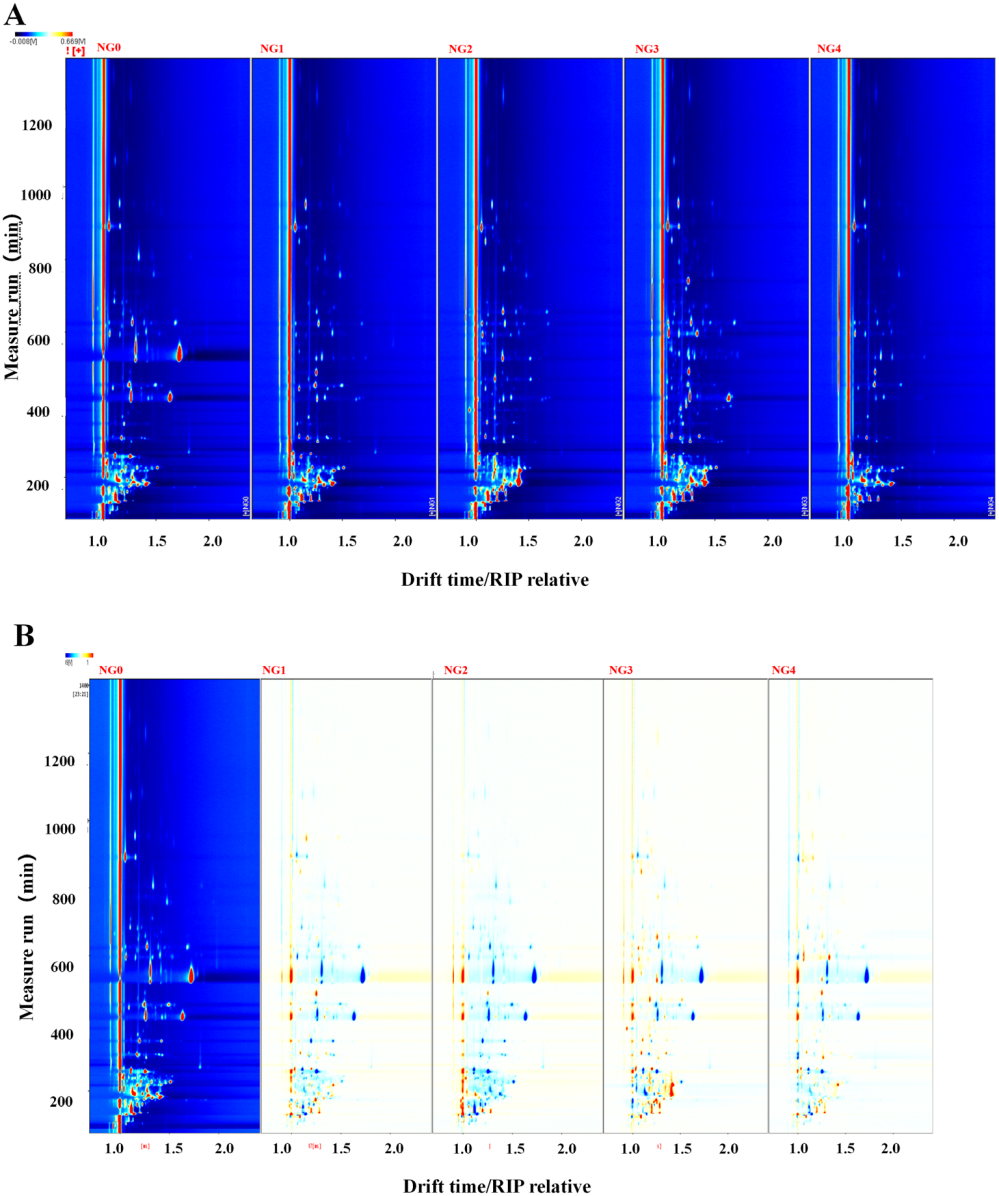
Composition spectrum (top view) and difference spectrum of volatile compounds in the sample.

Figure 2 shows the 2D spectrum (top view) and different spectra of the volatile compounds in the sample. Each bright spot in the spectrum represents a volatile compound, but some analytes with high proton affinity, such as monomers, dimers, and other different forms, may have more than one spot. The background of the entire figure is blue, and the red vertical line at the horizontal axis 1.0 is the RIP peak (reactive ion peak, normalized). The vertical axis represents the retention time (s) of the gas chromatogram, and the horizontal axis represents the relative migration time (normalized, a.u.). Each point on both sides of the RIP peak represents a volatile organic compound. The color represents the peak intensity of the substance, from blue to red; the darker the color, the greater the peak intensity. From Figure 2, we can see more clearly that its volatile compounds are mainly concentrated in the migration time of 1.0–1.5 in the abscissa and the retention time of 100–400 in the ordinate. The difference spectrum in Figure 2 is the difference analysis of the GC‐IMS spectrum (top view). Taking the fresh *B. edulis* sample as a reference, the comparison shows the differences in all volatile compounds in the steamed, fried, roasted, and boiled *B. edulis* samples. The red represents that the concentration of the substance in the sample is higher than the reference sample, while the blue represents that it is lower than the reference sample. The figure also shows that the content of volatile compounds in roasted and fried *B. edulis* is significantly higher than that in steamed and boiled *B. edulis*.

The results of two‐dimensional and three‐dimensional spectra of volatile compounds in roasted and fried *B. edulis* showed that roasted *B. edulis* has the highest volatile component content, while boiled *B. edulis* and steamed *B. edulis* have lower volatile component content. This is because after high‐temperature treatment, *B. edulis* will produce some special volatile flavor compounds. Although steamed *B. edulis* and boiled *B. edulis* are also heated at high temperatures, the heating temperature is lower than that of roasting and frying due to the presence of water. High temperature will cause the Maillard reaction of *B. edulis*, which will produce pyrazine and aldehyde compounds, thus producing the unique flavor of fried or roasted *B. edulis* (Low et al. 2007).

### 3.2 Effects of Different Cooking Methods of Boletus Eduli on Volatile Compounds

**FIGURE 3:**
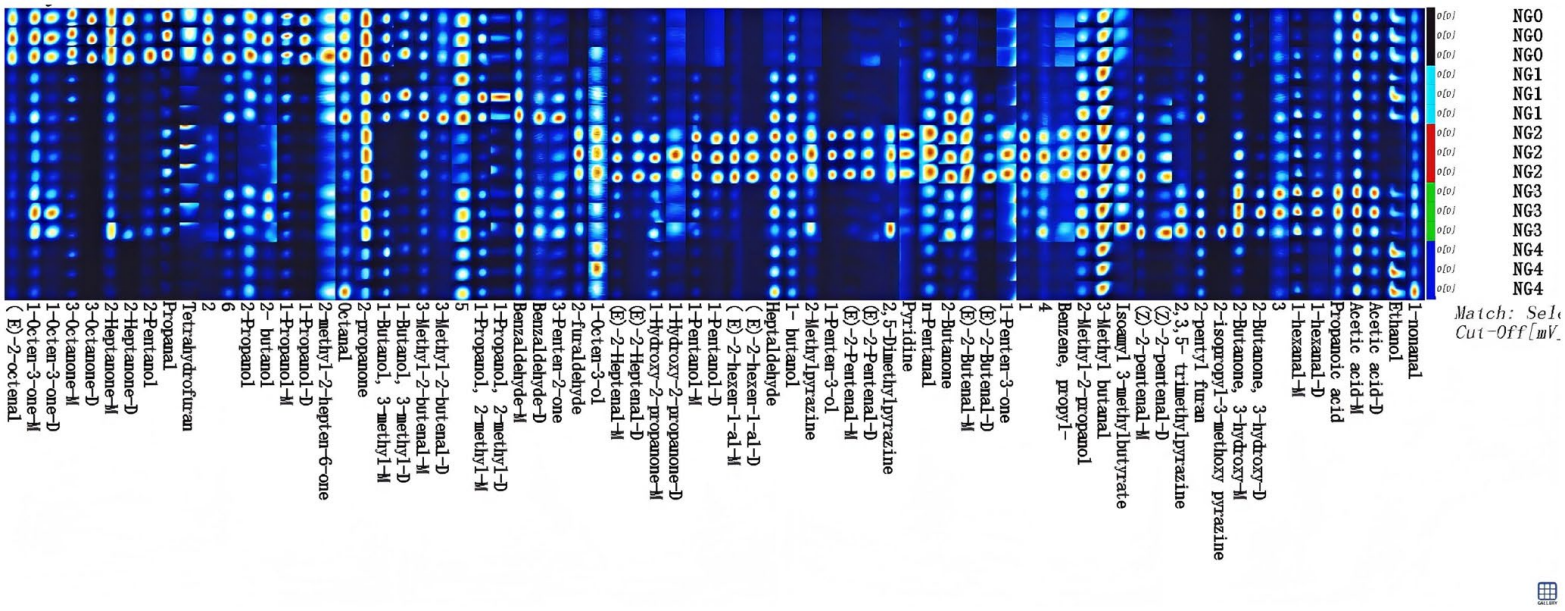
Fingerprint spectrum of sample gallery plot. *D*, dimer; *M*, monomer.

To more comprehensively characterize the variation patterns of volatile compounds in *B. edulis* under different cooking methods, a fingerprint map of volatile compounds was generated using a graphical plug‐in. In Figure 3, each row represents the volatile profile of one sample, and each column corresponds to the signal peak of a specific volatile compound across the various samples. The intensity of each signal peak reflects the concentration of the same compound across different sample groups: brighter colors indicate higher content, while darker colors denote lower levels. This visualization approach enables intuitive comparison of differences in the same volatile organic compound among various sample groups. Based on the results derived from Figure 3 and summarized in Table 1, a total of 49 volatile compounds were detected in the samples of *B. edulis* subjected to five different processing methods. These include 16 aldehydes, 12 alcohols, 10 ketones, 1 ester, 4 pyrazines, 2 furans, 2 acids, and 2 heterocyclic compounds. Table 1 provides detailed information for each compound, including name, CAS number, chemical formula, structural diagram, molecular weight, retention index, drift time, peak volume, and relative content.

**TABLE 1:** Detailed list of volatile compounds in the samples.

Count	Compound	CAS#	Formula	Structural type	MW	RI	Rt [sec]	Dt [a.u.]	Relative content 100%				
									NG0	NG1	NG2	NG3	NG4
1	Benzaldehyde‐M	C100527	C_7_H_6_O	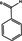	106.1	1501.9	955.585	1.15857	0.936699	1.134798	1.679928	2.758942	2.594969
2	Benzaldehyde‐D	C100527	C_7_H_6_O	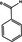	106.1	1502.5	956.841	1.47845	0.107618	0.133657	0.212529	0.376269	0.343335
3	Aceticacid‐M	C64197	C_2_H_4_O_2_	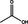	60.1	1470.2	888.993	1.0543	5.052227	5.930888	6.657623	8.111909	7.088200
4	Aceticacid‐D	C64197	C_2_H_4_O_2_	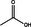	60.1	1472.1	892.762	1.1621	0.815666	0.874957	0.837085	0.851071	0.693835
5	2‐furaldehyde	C98011	C_5_H_4_O_2_	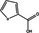	96.1	1450.6	850.043	1.09141	0.097201	0.214786	0.497033	0.902949	1.155300
6	Propanoic acid	C79094	C_3_H_6_O_2_	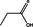	74.1	1553	1073.691	1.11439	0.435388	0.467046	0.478740	0.505384	0.445639
7	1‐Octen‐3‐ol	C3391864	C_8_H_16_O	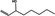	128.2	1458.9	866.377	1.16564	0.060202	0.084367	0.135770	0.176964	0.182664
8	(E)‐2‐octenal	C2548870	C_8_H_14_O	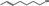	126.2	1429.3	809.836	1.3406	0.990100	0.381151	0.464069	0.548571	0.397888
9	1‐nonanal	C124196	C_9_H_18_O	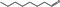	142.2	1400.5	758.322	1.48199	0.523483	0.650429	0.803746	1.003289	0.862175
10	2,3,5‐ trimethylpyrazine	C14667551	C_7_H_10_N_2_	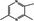	122.2	1419.7	792.234	1.16515	0.074579	0.099896	0.144870	0.232957	0.198280
12	2‐isopropyl‐3‐methoxy pyrazine	C25773404	C_8_H_12_N_2_O	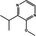	152.2	1390.8	741.692	1.25017	0.084649	0.112756	0.165962	0.257416	0.250944
13	1‐Octen‐3‐one‐M	C4312996	C_8_H_14_O	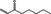	126.2	1316.3	625.922	1.28209	1.849850	1.872487	1.751441	1.878760	1.787702
14	1‐Octen‐3‐one‐D	C4312996	C_8_H_14_O	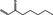	126.2	1316.3	625.922	1.68749	0.629111	0.574776	0.432458	0.303213	0.318833
15	1‐Hydroxy‐2‐propanone‐M	C116096	C_3_H_6_O_2_	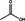	74.1	1316.3	625.922	1.05982	0.511937	0.614980	0.757596	0.986843	1.280236
16	1‐Hydroxy‐2‐propanone‐D	C116096	C_3_H_6_O_2_	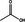	74.1	1315.8	625.157	1.23356	0.043367	0.043016	0.055840	0.064430	0.099104
17	2‐Butanone, 3‐hydroxy‐M	C513860	C_4_H_8_O_2_	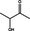	88.1	1294.2	594.547	1.06295	1.343815	1.330920	1.314044	0.973559	1.518513
18	2‐Butanone, 3‐hydroxy‐D	C513860	C_4_H_8_O_2_	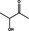	88.1	1294.9	596.077	1.33218	0.669945	0.651339	0.600214	0.387337	0.474755
19	2‐Methylpyrazine	C109080	C_5_H_6_N_2_	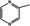	94.1	1270.8	549.396	1.09269	0.061785	0.109275	0.190922	0.341322	0.476905
20	3‐Octanone‐M	C106683	C_8_H_16_O	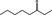	128.2	1262	533.325	1.3087	7.114241	6.456875	5.177087	2.836331	2.024129
21	3‐Octanone‐D	C106683	C_8_H_16_O	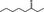	128.2	1261.2	531.795	1.72349	21.538401	18.047480	11.181412	0.995144	0.876390
22	2‐Heptanone‐M	C110430	C_7_H_1_4O	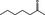	114.2	1190	417.858	1.26363	3.532997	3.379172	3.002142	2.300469	1.884301
23	2‐Heptanone‐D	C110430	C_7_H_14_O	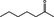	114.2	1190.4	418.486	1.63573	7.192315	6.186067	3.862516	0.814969	0.745131
24	1‐Butanol, 3‐methyl‐M	C123513	C_5_H_12_O	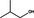	88.1	1217.5	458.693	1.24656	2.567520	2.468729	3.196674	3.729063	3.100080
25	1‐Butanol, 3‐methyl‐D	C123513	C_5_H_12_O	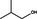	88.1	1216.2	456.808	1.4969	0.880974	0.826099	1.301231	1.377186	1.235237
26	3‐Methyl‐2‐butenal‐M	C107868	C_5_H_8_O	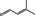	84.1	1208.4	444.872	1.0918	0.566299	0.680004	0.883923	1.420601	1.326134
27	3‐Methyl‐2‐butenal‐D	C107868	C_5_H_8_O	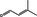	84.1	1209.7	446.756	1.36376	0.175552	0.176170	0.217486	0.341763	0.318775
28	1‐Penten‐3‐ol	C616251	C_5_H_10_O	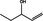	86.1	1167.1	384.962	0.93936	0.153711	0.177889	0.211124	0.254152	0.897890
29	(E)‐2‐Butenal‐M	C123739	C_4_H_6_O	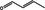	70.1	1059.1	267.102	1.03486	0.242943	0.356576	0.706311	1.436978	1.772028
30	(E)‐2‐Butenal‐D	C123739	C_4_H_6_O	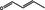	70.1	1058.3	266.421	1.20421	0.113044	0.122064	0.213581	0.592652	0.847814
31	(E)‐2‐Pentenal‐M	C1576870	C_5_H_8_O	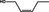	84.1	1140.7	350.218	1.10744	0.155990	0.171697	0.196233	0.229637	0.572338
32	(E)‐2‐Pentenal‐D	C1576870	C_5_H_8_O	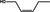	84.1	1142.3	352.261	1.36465	0.063055	0.071801	0.083743	0.065747	0.138363
33	(E)‐2‐hexen‐1‐al‐M	C6728263	C_6_H_10_O	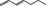	98.1	1226	472.165	1.18384	0.307144	0.385299	0.501421	0.671453	1.293723
34	(E)‐2‐hexen‐1‐al‐D	C6728263	C_6_H_10_O	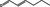	98.1	1225.2	470.803	1.52382	0.039078	0.054838	0.073161	0.100975	0.285838
35	2‐pentyl furan	C3777693	C_9_H_14_O	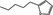	138.2	1238.1	491.922	1.25514	0.346407	0.760817	1.382188	2.978751	2.188028
36	1‐Pentanol‐M	C71410	C_5_H_12_O	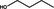	88.1	1260.6	530.755	1.25387	0.332278	0.496395	0.738134	1.193258	2.036167
37	1‐Pentanol‐D	C71410	C_5_H_12_O	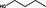	88.1	1258.7	527.348	1.52	0.086749	0.096265	0.113865	0.128439	0.322337
38	Pyridine	C110861	C_5_H_5_N	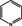	79.1	1188.5	415.62	1.02467	0.074597	0.099354	0.133844	0.187695	0.349110
39	(E)‐2‐Heptenal‐M	C18829555	C_7_H_12_O	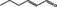	112.2	1337.6	657.018	1.2597	0.349458	0.472704	0.622381	0.929576	1.577410
40	(E)‐2‐Heptenal‐D	C18829555	C_7_H_12_O	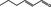	112.2	1336.8	655.831	1.67642	0.079913	0.094388	0.120762	0.154978	0.308234
42	2,5‐Dimethylpyrazine	C123320	C_6_H_8_N_2_	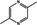	108.1	1331.6	648.111	1.11814	0.034505	0.042061	0.055393	0.074411	0.175859
43	2‐Isoamyl 3‐methylbutyrate	C659701	C_10_H_20_O_2_	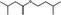	172.3	1312.7	620.794	1.45852	0.084649	0.112756	0.165962	0.257416	0.250944
45	Octanal	C124130	C_8_H_16_O	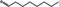	128.2	1298.2	600.603	1.40921	0.559046	0.634909	0.705405	0.908453	0.752838
46	1‐Propanol, 2‐methyl‐M	C78831	C_4_H_10_O	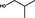	74.1	1104.6	307.809	1.17366	1.402463	1.491012	2.138241	2.783372	2.354119
47	1‐Propanol, 2‐methyl‐D	C78831	C_4_H_10_O	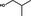	74.1	1105.3	308.635	1.36789	0.105867	0.101555	0.232456	0.343974	0.319318
48	(Z)‐2‐pentenal‐M	C1576869	C_5_H_8_O	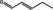	84.1	1109.6	313.433	1.09478	0.293234	0.396009	0.622312	1.129099	1.409853
49	(Z)‐2‐pentenal‐D	C1576869	C_5_H_8_O	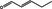	84.1	1108.5	312.125	1.35157	0.057597	0.067817	0.137301	0.302286	0.401009
50	1‐hexanal‐M	C66251	C_6_H_12_O	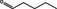	100.2	1094.3	297.077	1.26696	0.431754	0.651097	0.928001	1.363625	1.345807
51	1‐hexanal‐D	C66251	C_6_H_12_O	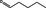	100.2	1095.5	298.229	1.56745	0.075179	0.100442	0.137896	0.214227	0.210631
52	1‐ butanol	C71363	C_4_H_10_O	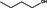	74.1	1154.1	367.507	1.18266	0.302592	0.426951	0.642739	0.921253	0.968908
53	3‐Penten‐2‐one	C625332	C_5_H_8_O	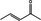	84.1	1139.5	348.783	1.07348	0.067315	0.087801	0.153564	0.308406	0.284837
54	2‐Pentanol	C6032297	C_5_H_12_O	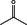	88.1	1136.9	345.507	1.20263	0.867940	0.905500	0.923429	0.298449	0.280526
55	Benzene, propyl—	C103651	C_9_H_12_	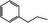	120.2	1196.6	427.424	1.15603	0.093640	0.096901	0.109008	0.118126	0.198226
57	2‐propanone	C67641	C_3_H_6_O	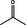	58.1	842.5	147.21	1.1152	11.005650	11.839754	12.979273	15.238032	15.722403
58	Propanal	C123386	C_3_H_6_O	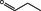	58.1	798	130.92	1.14755	3.386184	3.016416	2.347127	1.211816	1.797774
59	Ethanol	C64175	C_2_H_6_O	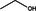	46.1	947.1	193.957	1.13194	4.380332	4.720670	6.213416	7.331588	5.492331
60	3‐Methyl butanal	C590863	C_5_H_10_O	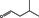	86.1	917.6	179.437	1.40971	3.502633	4.589573	5.628931	7.305787	6.723786
61	2‐Butanone	C78933	C_4_H_8_O	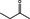	72.1	907	174.479	1.24907	0.705230	0.891876	1.304787	2.568142	3.074099
62	Tetrahydrofuran	C109999	C_4_H_8_O	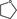	72.1	874.8	160.314	1.23011	0.542776	0.507392	0.414529	0.256882	0.315269
63	2‐Methyl‐2‐propanol	C75650	C_4_H_10_O	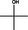	74.1	914.6	178.021	1.32939	1.184458	1.460706	1.913595	2.796608	2.929200
64	2‐Propanol	C67630	C_3_H_8_O	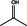	60.1	939.4	190.062	1.22899	0.721331	0.765766	0.872187	0.932162	0.662189
65	n‐Pentanal	C110623	C_5_H_10_O	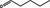	86.1	990.9	217.685	1.42198	0.403165	0.926816	1.442750	2.391076	3.176733
68	2‐ butanol	C78922	C_4_H_10_O	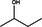	74.1	1021.1	238.088	1.15421	0.433790	0.475196	0.434267	0.429248	0.373825
69	1‐Penten‐3‐one	C1629589	C_5_H_8_O	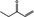	84.1	1033.7	247.321	1.07758	0.067281	0.074189	0.085804	0.090459	0.124268
70	1‐Propanol‐M	C71238	C_3_H_8_O	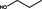	60.1	1047.6	257.989	1.11254	3.293667	2.982155	2.804222	2.210602	2.048019
71	1‐Propanol‐D	C71238	C_3_H_8_O	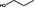	60.1	1047.6	257.989	1.25719	3.013095	2.575631	1.900093	0.462884	0.499552
72	2‐methyl‐2‐hepten‐6‐one	C110930	C_8_H_14_O	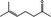	126.2	1354.7	683.192	1.18018	0.136278	0.168743	0.200447	0.216521	0.160920
73	Heptaldehyde	C111717	C_7_H_14_O	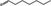	114.2	1191.2	419.713	1.33905	0.131906	0.286524	0.513244	0.981320	1.050780

As illustrated in Figure 3, the fresh porcini sample (NG0) exhibited markedly higher signal intensities for 2‐octenal, 1‐octen‐3‐one, and 3‐octanone compared to the other treatment groups. Relative to the fresh group, the steamed samples (NG1) showed a pronounced reduction in the signal intensities of 2‐octenal, 1‐octen‐3‐one, and 3‐octanone, whereas the signals corresponding to 1‐octen‐3‐ol and heptanal were significantly enhanced. In the fried group (NG2), elevated signal levels were observed for multiple compounds, including 2‐furaldehyde, 1‐octen‐3‐ol, 2‐heptenal, 1‐hydroxy‐2‐propanone, amyl alcohol, 2‐ethylene‐1‐aldehyde, 2‐methylpyrazine, 1‐penten‐3‐ol, 2‐pentenal, 2,5‐dimethylpyrazine, pyridine, valeraldehyde, 1‐pentenetrione, and pentenal, all of which were significantly higher than those in the other cooked groups. The roasted *B. edulis* (NG3) displayed strong signals for compounds such as 1‐octen‐3‐one, 2‐methyl‐2‐hepten‐6‐one, 2‐propanone, 2‐butanone, 1‐propanol, benzaldehyde, 3‐penten‐2‐one, trimethylpyrazine, and 2‐pentylfuran. Meanwhile, in the boiled samples (NG4), prominent signals were detected for 2‐propanone, 1‐octen‐3‐ol, heptanal, and 3‐methylbutanal.

The aroma threshold of 1‐octen‐3‐ol is low (14–25 μg/kg), and it exhibits the characteristic scents of mushrooms, lavender, roses, and hay. In contrast, the aroma threshold of 1‐octen‐3‐one is even lower (0.05–4 μg/kg), imparting a robust earthy and mushroomy aroma. Due to the presence of unsaturated double bonds in both compounds, which render their chemical properties unstable, the processing techniques applied to mushrooms can influence the content of these compounds, thereby impacting the overall aroma profile of mushrooms (Muteliefu et al. 2001). As illustrated in Figure 3, the signal intensities of 1‐octen‐3‐one and 1‐octen‐3‐ol in *B. edulis* undergo significant alterations under different cooking methods. The signal intensity of 1‐octen‐3‐one was most intense in fresh *B. edulis* (NG0) and weakest in the boiled group. In steamed (NG1) and fried groups, the signal of 1‐octen‐3‐one showed varying degrees of reduction, while in the roasted group (NG3), its intensity remained comparable to that of the fresh sample. In contrast, the signal intensity of 1‐octen‐3‐ol was higher in all cooked treatment groups compared to the fresh group (NG0), with the highest intensity observed in the fried samples (NG2). This indicates that various cooking approaches can substantially modify the characteristic mushroom flavor of *B. edulis*.

Since aldehyde compounds possess a low odor threshold and potent odor characteristics, they exert a crucial influence on the overall aroma of food (Huang et al. 2022). From Figure 3, it can be observed that compared to other treatment groups, the fried group exhibited a significant increase in both the variety and relative content of aldehydes, including 2‐Furfural, 2‐Heptenal, 2‐Ethenyl‐1‐al, 2‐Pentenal, Pentanal, and Pentenal. This is likely because triglycerides in cooking oil are prone to undergo thermal degradation and hydrolysis under frying conditions, generating free fatty acids. These free fatty acids can then form different types of hydroperoxides through reactions at the carbon–carbon double bonds, such as homolytic β‐scission, keto‐enol tautomerism, or isomerization (Yin et al. 2011). Subsequently, each hydroperoxide can decompose into low‐molecular‐weight volatile compounds (e.g., aldehydes, hydrocarbons, ketones, alcohols, and carboxylic acids) (Zhang et al. 2015). Aldehydes generated during the frying process, in conjunction with other volatile compounds, contribute to a desirable complex flavor profile in fried *B. edulis*. Benzaldehyde, characterized by its sweet almond‐like aroma, is a derivative of benzoic acid. This structural compound has been demonstrated to contribute to the distinct mushroom aroma (Li et al. 2023). Compared with the fresh group, the signal intensity of benzaldehyde significantly increased in the roasted groups. This enhancement is likely attributed to the degradation of benzoic acid into benzaldehyde during the roasting process (Cheng et al. 2022). In summary, aldehydes play a pivotal role in determining the odor characteristics of *B. edulis*. The substantial generation of aldehydes during cooking significantly enhances the overall flavor profile of the mushroom. During frying, some aldehydes may originate from the thermal decomposition and hydrolysis of cooking oils, while roasting effectively promotes the release of endogenous aldehydes from *B. edulis* itself through Maillard reaction and lipid oxidation pathways.

Furans, pyrazines, and pyridines are widely recognized as characteristic aroma compounds that occur in trace amounts within both frying oil and fried food products. Urfural was predominantly detected in the fried group, exhibiting sweet, woody, almond, and toasty aromas. It is speculated that furfural may originate from the degradation products of hydrogen peroxide and carbohydrates; however, its presence in edible fungi has not been previously reported (Yu et al. 2021). The content of pyrazine increased after frying or roasted cooking, which may have occurred due to the thermal degradation of threonine and serine. The occurrences and concentrations of these compounds are mainly related to frying time, temperature, and the complexity of food ingredients (especially amino acids and reducing sugars). Furan and pyrazine compounds exhibited markedly stronger signals in the fried and roasted groups. These compounds typically impart characteristic aromas—including sweet, burnt, and caramel‐like notes—to deep‐fried foods (Klis et al. 2011). *B. edulis* has a relatively high content of threonine (Guo et al. 2018). Therefore, furan and pyrazine compounds may originate from the high‐temperature reactions of threonine during frying and grilling.

### 3.3 PCA Analysis

PCA, also referred to as principal component analysis, is a dimensionality reduction technique applicable to multiple variables. As an unsupervised learning method, it facilitates a broad assessment of overall statistical discrepancies among sample groups, as well as the degree of parallelism between samples within the same group.

**FIGURE 4:**
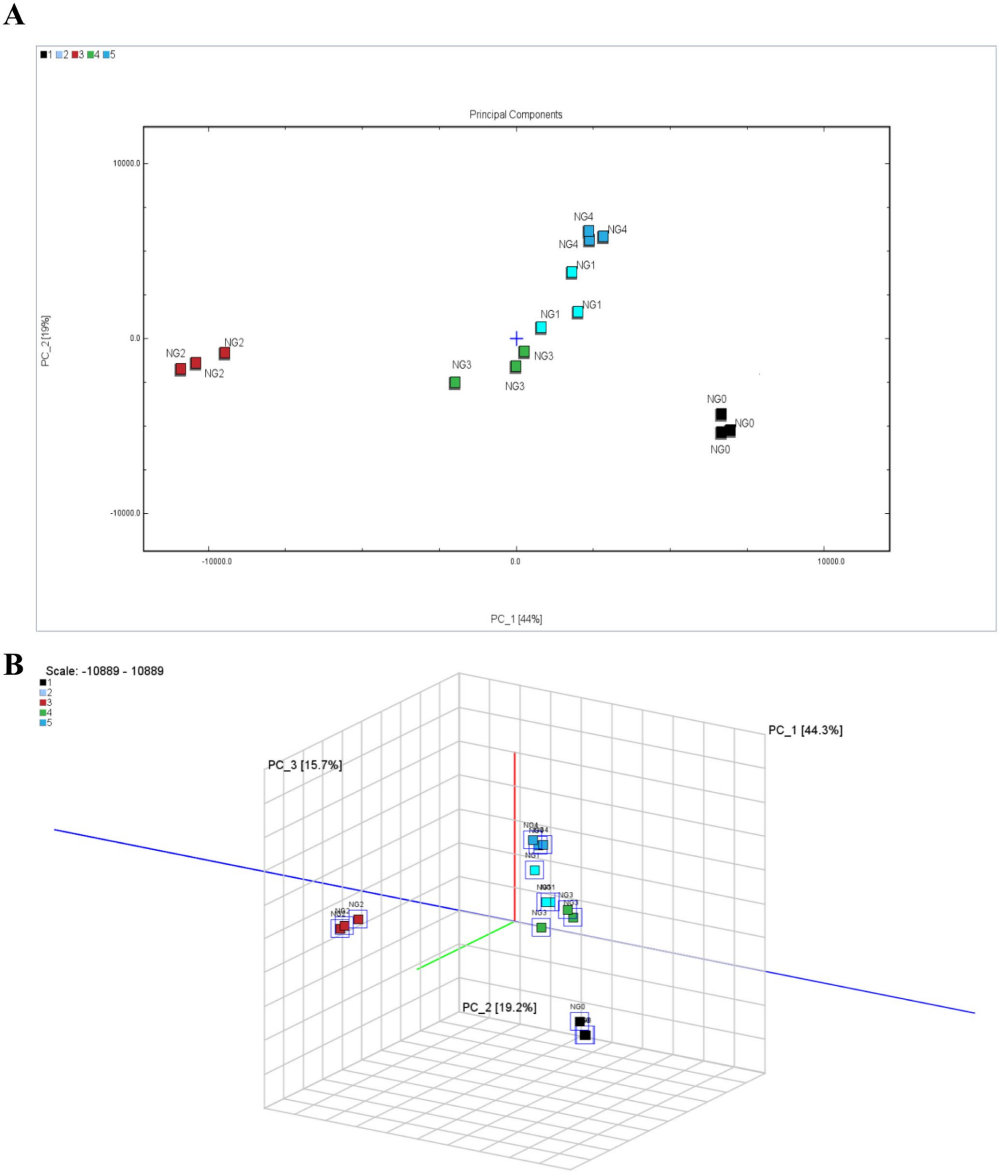
PCA score plot of volatile compounds in samples.

PCA was applied to reduce the dimensionality and recognize patterns in the volatile compound data. By analyzing the clustering and separation of samples in the principal component score plot, the overall flavor profile changes caused by different cooking methods were systematically compared and visualized. This was combined with the flavor fingerprint plot for the qualitative and relative quantitative analysis of key compounds contributing to the differences. The principal component score plot is depicted in Figure 4. Notably, the cumulative contribution rates of the first two principal components (PC1 and PC2) and the first three principal components (PC1, PC2, and PC3) reach 64% and 80%, respectively. These results indicate a strong correlation among variables, a high explanatory power for the original variables, and the retention of relatively comprehensive original information. As illustrated in the figure, parallel samples are closely clustered, demonstrating excellent reproducibility. Moreover, the separation between different samples is distinct, highlighting significant inter‐group differences. The disparities among samples revealed by PCA are consistent with the intuitive observations from the fingerprint spectra, validating the effectiveness of this analytical approach. As demonstrated in the figure, the coordinate axes effectively differentiated the four sample groups, suggesting that the flavor profiles were substantially influenced by the cooking methods applied.

In the Figure 4, the closer the samples are to each other, the higher the similarity in their aroma compositions and relative contents. The characteristic flavors of processed *B. edulis* and fresh *B. edulis* occupy non‐overlapping quadrants, which indicates that the volatile flavor profile of *B. edulis* undergoes significant alterations after being subjected to different cooking methods. Within food materials, the high temperature, oxygen, and moisture associated with cooking are capable of inducing various biological and chemical reactions (Goswami and Manna 2020). The cooking process can potentially influence the overall aroma characteristics of processed foods via the Maillard reaction (Shakoor et al. 2022), lipid oxidation, amino acid degradation, and their combined reactions (Flaskerud et al. 2017; Wen Li et al. 2022). These chemical transformations collectively account for the differences in the volatile flavors of *B. edulis* following the four cooking methods.

### 3.4 E‐Nose Analysis

**FIGURE 5:**
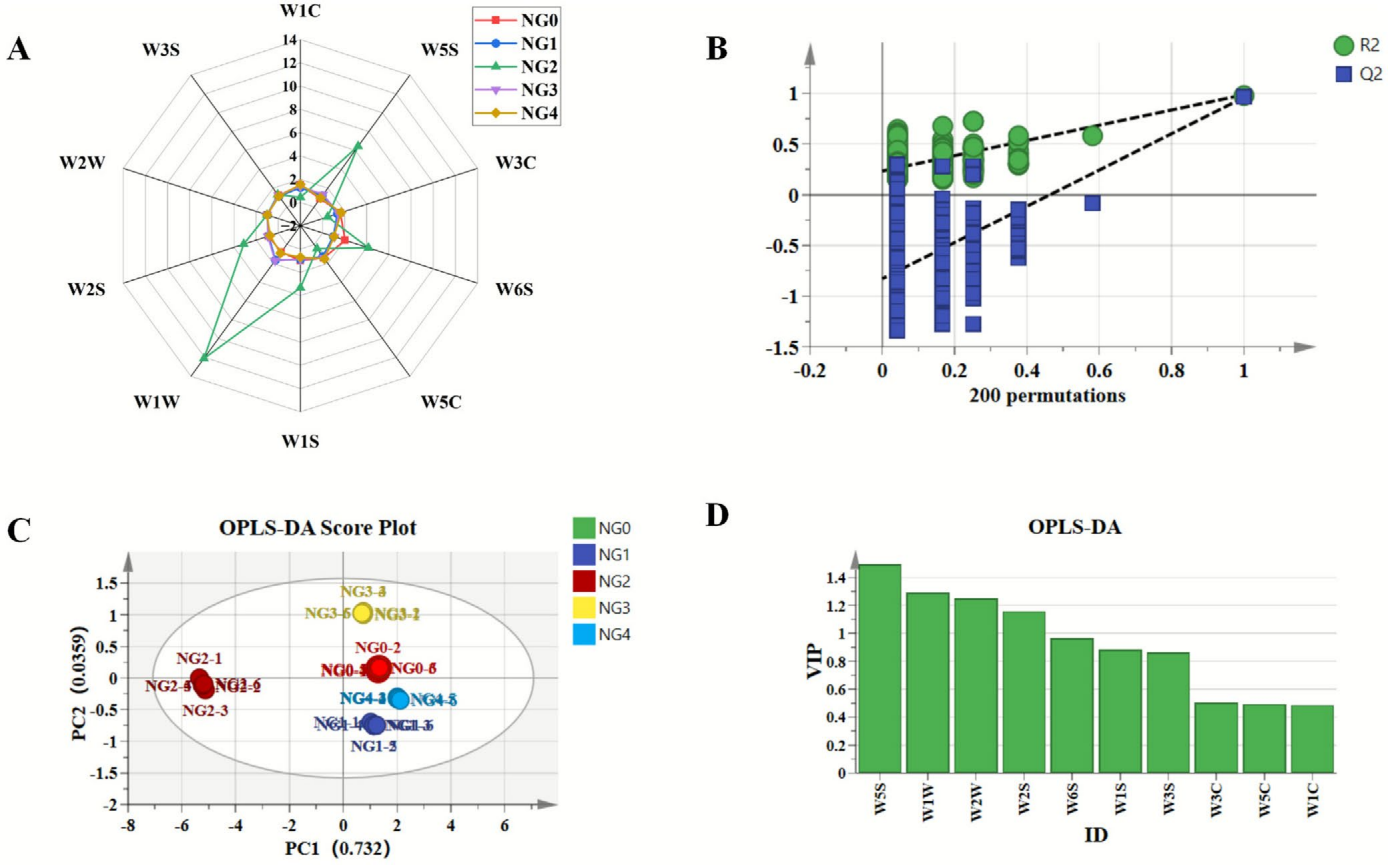
E‐nose analysis of button *Boletus edulis* with different cooking methods. (A) E‐nose radar diagram; (B) Two hundred permutation test chart of OPLS‐DA model; (C) OPLS‐DA analysis of E‐nose; (D) VIP values of OPLS‐DA model.

As an electrochemical sensing system that simulates human olfaction, the electronic nose is characterized by its ability to generate specific responses to complex volatile organic compounds (VOCs) in food via an array of gas sensors. These responses are subsequently processed using pattern recognition algorithms—such as principal component analysis and discriminant factor analysis—to facilitate rapid qualitative or quantitative flavor analysis (Zhou et al. 2015). As illustrated in Figure 5A, the response values of the 10 sensors to *B. edulis* differed across cooking methods. The highest response was observed with sensor W1W, followed by W5S, W6S, W1S, and W2S, indicating that sulfides, nitrogen oxides, hydrides, alkanes, and alcohols constitute the major volatile compounds of this mushroom. Meanwhile, GC‐IMS analysis revealed comparatively strong signals of nitrogen oxides, aldehydes, and alcohols in fried samples (NG2). This outcome may be attributed to the Maillard reaction: under high‐temperature conditions, polysaccharides and proteins in *B. edulis* undergo chemical transformations that generate abundant aromatic compounds during frying. (Deng et al. 2024).

To further explore the impact of various cooking methods on the odor profile of *B. edulis*, an OPLS‐DA model was developed in this study, with the analytical outcomes illustrated in Figure 5B–D. As indicated in Figure 5C, the cumulative contribution rate of PC1 and PC2 reached 76.79%, suggesting that these two principal components effectively capture the majority of volatile odor characteristics present in the samples. According to the Orthogonal Projections to Latent Structures‐Discriminant Analysis (OPLS‐DA), the explanatory power of the independent variables (*R* ^2^ *X*) was 0.999, the dependent variable fitting index (*R* ^2^Y) attained 0.989, and the predictive capability (*Q* ^2^) reached 0.971. With both *R* ^2^ and *Q* ^2^ values surpassing 0.5, the model demonstrates both high explanatory power and predictive reliability (Yun et al. 2021). As presented in Figure 5B, 200 permutation tests revealed that the *Q* ^2^ regression line intersects the vertical axis below zero, indicating the absence of overfitting and confirming the validity of the model. These results are therefore deemed suitable for discriminant analysis of cooking methods applied to *B. edulis*. In Figure 5C, samples from the fresh group (NG0), steaming group (NG1), boiling group (NG4), and baking group (NG3) are distributed on the positive semi‐axis of PC1, while the samples subjected to frying are entirely distributed on the negative semi‐axis of PC1. There is a good degree of separation between different treatment groups with favorable repeatability, which indicates that electronic nose technology can effectively distinguish *B. edulis* treated with different cooking methods, highlighting that cooking processes lead to significant changes in the overall distribution of volatile aromas in *B. edulis*. Furthermore, the *B. edulis* samples treated with the four cooking methods are successfully differentiated in the score scatter plot. Distributed along the negative semi‐axis of PC2 in close proximity, the boiling and steaming groups exhibit a similar spatial arrangement, suggesting potential similarities in certain flavor compounds between these two sample types. In contrast, the samples in the baking group are concentrated on the positive semi‐axis of PC2, indicating that their flavor characteristics are significantly different from those of the other two groups. The reason may be as follows: during the baking process, the *B. edulis* undergoes high‐temperature drying, leading to rapid evaporation of moisture and significant shrinkage of the mushroom surface, which may be more conducive to the retention and release of volatile compounds; whereas the steaming and boiling treatments may cause partial dissolution of flavor compounds in water, resulting in losses (Zhang et al. 2023; Zhou et al. 2022). Notably, the fried samples (NG2) were completely separated on the negative semi‐axis of PC1, indicating their unique flavor profile. This macroscopic discrimination was strongly supported by the microscopic compound data from GC‐IMS. The GC‐IMS flavor fingerprint (Figure 3) and qualitative results (Table 1) demonstrated that fried and roasted (NG3) samples generated a substantial number of characteristic volatile compounds. These included compounds significantly enriched in fried samples, such as 2‐furaldehyde, 2‐methylpyrazine, pyridine, and various aldehydes, as well as compounds abundant in roasted samples, like 2‐pentylfuran, trimethylpyrazine, and benzaldehyde. These unique compounds, generated primarily via the Maillard reaction and lipid oxidation, collectively constitute the overall flavor signals perceived by the E‐nose, distinguishing them from other samples.

Figure 5D further displays the variable importance in projection (VIP) values derived from the OPLS‐DA model, which quantifies the response magnitude of each sensor and reflects the relative contribution of individual variables. A higher VIP value (VIP > 1) signifies a greater abundance of the corresponding substance in the flavor profile of *B. edulis*. All four sensors—W5S, W1W, W2W, and W2S—exhibited VIP values exceeding 1, indicating that these four odor categories are prominent in the *B. edulis* and that the corresponding sensors play a significant role in the OPLS‐DA discrimination. The significance of the W5S sensor stemmed from nitrogen‐containing heterocyclic compounds, such as pyrazines and pyridines, generated in abundance during the Maillard reaction in fried and roasted samples. Furthermore, the high VIP value of the W2S sensor aligned with the trends observed via GC‐IMS for significantly increased levels of alcohols (e.g., 1‐octen‐3‐ol) and aldehydes after heat treatment. This correspondence strongly confirms that the overall flavor discrimination by the E‐nose is based on the specific responses of its sensors to key classes of flavor compounds in the samples.

### 3.5 Sensory Evaluation Analysis

**FIGURE 6:**
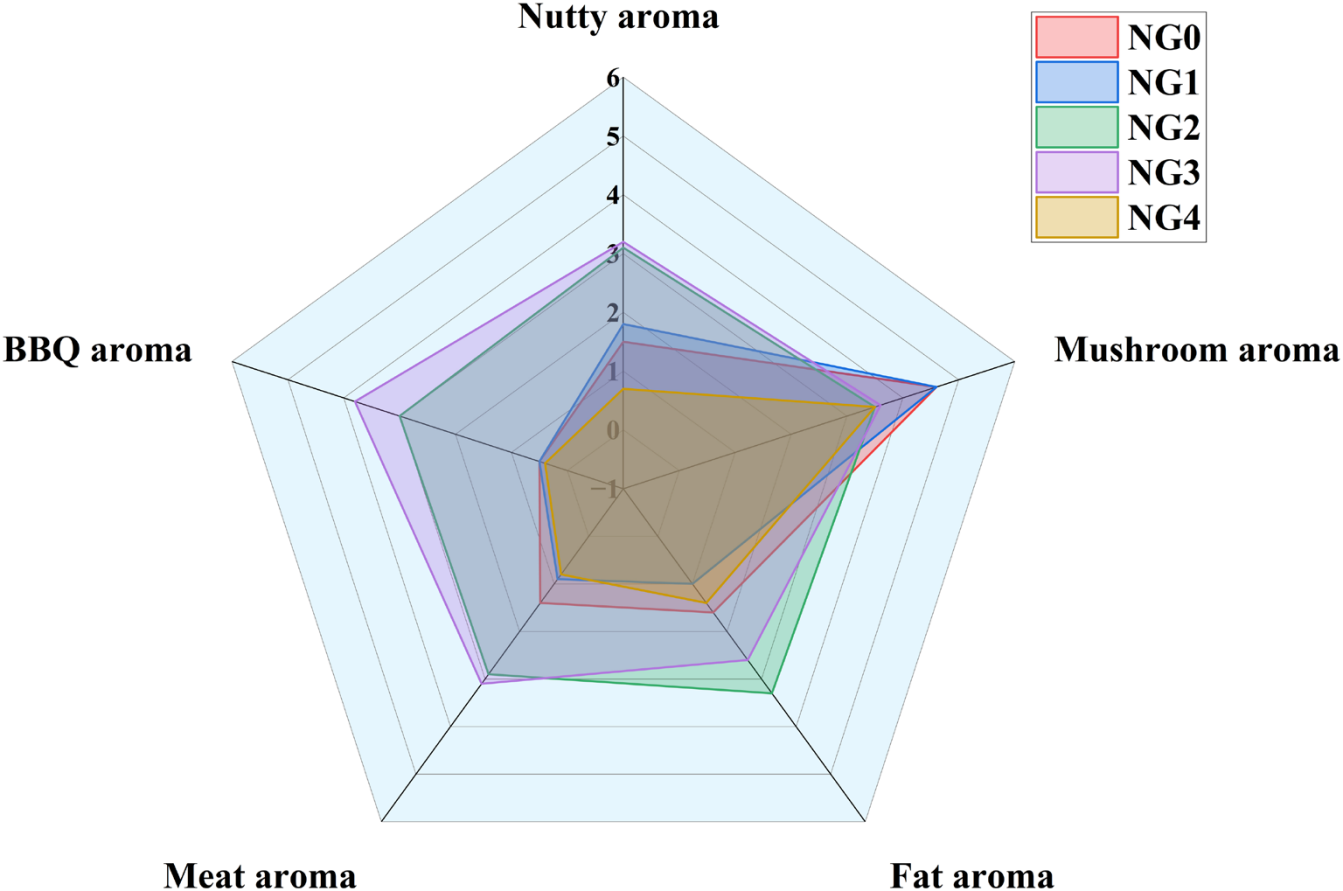
Sensory evaluation radar chart.

The radar chart in Figure 6 illustrates the sensory evaluation of five differently processed *B. edulis* samples. The results indicate that fresh (NG0), roasted (NG3), and boiled (NG4) *B. edulis* exhibited a pronounced mushroom flavor during the sensory assessment. The intense mushroom flavor in *B. edulis* is primarily contributed by eight‐carbon compounds, including 1‐octen‐3‐ol and 1‐octen‐3‐one (Zhang et al. 2018). According to the GC‐IMS analysis, the signal intensity of 1‐octen‐3‐one was more intense in both fresh and roasted samples compared to other treatment groups, which aligns with and is corroborated by their higher sensory evaluation scores. Notably, the fried (NG2) and roasted (NG3) *B. edulis* samples received the highest scores in flavor evaluation. According to GC‐IMS analysis, this can be primarily attributed to pyrazines, pyridines, and aldehydes generated through the Maillard reaction during high‐temperature processing, which collectively contribute to the characteristic rich grilled aroma. Comprehensive sensory evaluation further confirmed that both fried (NG2) and roasted (NG3) samples not only achieved higher overall scores but also exhibited more balanced flavor profiles. These results suggest that frying and roasting could potentially represent optimal cooking methods for enhancing the flavor quality of *B. edulis* (Figure 6).

From the perspective of flavor profiles, those of fresh and boiled *B. edulis* products are relatively similar. This similarity can be attributed to the fact that boiling preserves most of the flavor compounds of *B. edulis* with minimal chemical alteration. Consequently, aside from some flavor compounds that dissolve and are lost in the cooking water, the remaining characteristic flavor compounds in the boiled samples closely resemble those in fresh *B. edulis*.

## 4 Conclusion

This study investigated the variations in volatile flavor compounds of *B. edulis* under diverse cooking methods. Through comparative analysis, it was demonstrated that the cooking method exhibits a complex relationship with both the composition and concentration of volatile flavor compounds in *B. edulis*. Furthermore, marked differences were observed in the types and levels of aroma compounds across various cooking techniques, each contributing unique sensory attributes to the final product. The flavor of boiled *B. edulis* closely resembled that of fresh specimens; however, baking yielded the most intense and appealing aroma. E‐2‐octenal, 1‐octen‐3‐one, 3‐octanone, 2‐heptanone, propanol, 2‐butanol, isopropanol, and 2‐pentanol were identified as the predominant volatile flavor compounds in fresh, steamed, and boiled *B. edulis*. In contrast, under the higher‐temperature cooking methods of frying and baking, aldehydes and pyrazines became the dominant volatile flavor compounds. A comparative analysis of the GC‐IMS and electronic nose results demonstrated that both techniques effectively distinguished *B. edulis* samples subjected to different cooking methods. Notably, the fried samples exhibited distinct separation from other treatment groups in both analytical approaches, indicating consistent and pronounced flavor profile alterations induced by frying. Nevertheless, the inability to establish statistically significant correlations between the two detection methods represents a limitation of this study. Future research must employ more advanced chemometric tools—such as gas chromatography‐olfactometry mass spectrometry or selective sensor arrays—to decipher the complex relationships between specific volatile compounds and electronic nose sensor responses.

The results demonstrate that cooking method is a critical factor determining the flavor profile of *B. edulis*. However, the specific mechanisms and reaction pathways governing flavor formation in *B. edulis* under different cooking conditions remain incompletely understood. Future research should focus on elucidating the precise chemical transformations involved, particularly through targeted investigation of key flavor precursors and systematic monitoring of reaction kinetics across varied thermal processing parameters.

These findings offer a theoretical foundation for flavor regulation during the cooking of *B. edulis*, provide data support for the enhancement and innovation of Boletus‐based dishes, and furnish technical assistance for the development of *B. edulis* products.

## Author Contributions

**Xin Wu:** funding acquisition, methodology, and writing‐original draft; **Jingfa Wang:** formal analysis and validation; **Huizhen Liu** and **Fanjun Sun:** investigation; **Fucan Liu:** software; **Jing He:** validation; **Furong Tian:** project administration; **Chunxia Gan:** writing – review and editing.

## Funding

This work was supported by Research Project for Introduced Talents of Kunming University (YL23042); Scientific Research Fund project of Yunnan Province Department of Education (2023J0825 and 2024J078); Science and Technology Program for Plateau‐Featured Agriculture (202502AE090041).

## Ethics Statement

The study was approved by the Ethics Committee of Kunming University (Approval No. KMU2025‐017).

## Consent

Written informed consent was obtained from all study participants.

## Conflicts of Interest

The authors declare no conflicts of interest.

## Data Availability

The authors have nothing to report.
